# Composition Optimisation of Selected Waste Polymer-Modified Bitumen

**DOI:** 10.3390/ma15248714

**Published:** 2022-12-07

**Authors:** Grzegorz Mazurek, Juraj Šrámek, Przemysław Buczyński

**Affiliations:** 1 Department of Civil Engineering and Architecture, Kielce University of Technology, Al. Tysiąclecia Państwa Polskiego 7, 25-314 Kielce, Poland; 2 Department of Construction Management, University of Zilina, Univerzitna 8215/1, 01001 Zilina, Slovakia

**Keywords:** waste plastomer, composition optimisation, rheology, modified bitumen, Plackett–Burman experimental design

## Abstract

Waste plastomer disposal is currently a major challenge facing modern economies. This article reports on a study and analysis regarding the implementation of plastomers into bitumen, with a special focus on the influence of mixing process factors. Two plastomers were selected for analysis, PP and PET, and two bitumen types, 20/30 and 70/100, were modified. Determination of the basic characteristics, such as penetration, softening temperature, cohesion energy, and Fraass temperature, was complemented with advanced multiple-stress creep recovery (MSCR) rheological testing. The entire experimental process followed the Plackett–Burman design. Rheological effects of modified bitumen were evaluated using the generalized Maxwell model. Microstructural analysis with epi-fluorescence microscopy showed the ability of plastomer-modified bitumen to obtain a fine-grained structure with a particle size of <10 μm. In addition, creep susceptibility (Jnr) was found to be statistically significantly dependent on the polymer type and particle size, rotational speed, and bitumen type. In turn, the particle dispersion structure in the bitumen matrix significantly depended on the rotational speed, plastomer particle size, and mixing temperature. Ultimately, the process of bitumen 70/100 modification was optimized. It was demonstrated, following the experimental design, that by using fine-grained PP for a temperature of 160 °C, rotational speed of about 6300 rpm and time of 105 min, it is possible to obtain modified bitumen with rheological properties very similar to those of modified bitumen PmB 45/80-55.

## 1. Introduction

Waste materials is a term describing materials generated or discarded during the manufacturing process, post-consumed waste, and pre-sorted (segregated) fractions of municipal waste (non-processed) unsuitable for direct use in industrial processing. Waste disposal policies have attracted the attention of researchers in terms of waste applicability to composites that have a structure of concrete. Examples of their application to produce good quality construction products include the use of waste glass as a replacement for a certain amount of cement [1] or the utilisation of coal incineration ash [2]. Additionally worthy of notice are fibres derived as waste from turning processes [3] or from used tyres [4].

Road engineering is another very important beneficiary of waste disposal technologies, where waste materials can be used to modify the physical and mechanical characteristics of base course layers [5,6,7] or applied as a substitute material for aggregate (recycled aggregates, blast furnace slag, fly ash) [8,9]. Another application allowing a reduction of the use of virgin aggregate is the implementation of dry-process polymer waste to significantly improve the fatigue life and rutting resistance of mineral-bituminous composites [10].

The modification with waste materials can also apply to bituminous binders. Dosing effectiveness depends primarily on the mixing process taking into account polymer and bitumen properties, e.g., solubility [11]. Historical perspective and the literature review on the stability of bitumen and polymer (waste polymer) modifications are covered in the extensive publication [12]. It is known that bitumen binder is a waste material derived from petroleum distillation. Bitumen binder is a waste material recycled during petroleum distillation. It is a thermoplastic material, which determines the durability of mineral and bitumen mixes [13]. The rheological properties of mineral and bitumen mixes depend mainly the used bitumen’s properties [14]. Most defects that occur in the pavement are caused by high and low temperatures, cyclic loading, and bitumen ageing. The bitumen’s low quality is, therefore, a reason for additional expenditure. The most popular method of improving bitumen’s rheological and functional properties, thereby providing a high-quality bitumen binder, is to introduce polymer into the bitumen’s matrix [14,15]. The most common method of modifying bitumen is to use elastomers, e.g., SBS [16]. Its introduction into the bitumen’s structure substantially expands the viscoelastic range of the base bitumen by ensuring its high utility value at low temperatures [17,18]. On the other hand, the introduction of plastomer-group polymers substantially improves its permanent deformation resistance [19]. When modifying bitumen with plastomer, the polymer should be selected in order to ensure that its softening point is below the component mixing temperature, usually in the range of 160–170 °C [20,21]. When using hard polymer, its presence raises the mineral and bitumen mix’s softening point, lowers the stability and rutting resistance, as well as lowers its low temperature resistance [22].

Nevertheless, the final properties of a polymer-modified bitumen depend on the type and properties of the added polymer, bitumen, their ratio and, more importantly, the mixing process [23]. The polymer’s reactivity and chemical structure affects its compatibility with bitumen, thereby affecting the mix’s quality [24]. During the bitumen’s modification with plastomer, it is possible that the components will become segregated due to the polymer’s high molecular weight, polarity or an insufficient maltene fraction in the bitumen [25]. The mixing process itself can also be disrupted by a high mixing temperature maintained for a prolonged time, thereby causing additional bitumen ageing resulting from the maltene fraction’s degradation or even polymer oxidation [26]. The effect is a quick loss of stability during the mix’s storage. A certain solution for the low storage stability is to reduce the share of non-polar polymer chains by using, e.g., butyl acrylate or reactive polymer [27,28]. On the other hand, bitumen modification using plastomer provides additional polar groups, due to the higher mixing temperature than when using regular elastomer, which results from the ageing process, thereby causing increased compatibility of bitumen and polymer [29]. Therefore, the modification process is a very important factor that must be observed when homogenising bitumen and plastomer.

There are many methods of recycling plastomer, including storage, sorting, and grinding. There are also chemical methods in the form of pyrolysis and gasification. Nevertheless, the most popular is the aforementioned mechanical method that provides a pellet-type material with varied granulation [30]. Many plastomers are available in the market. Among those, it is possible to distinguish two popular types available in large quantities. The first type is polypropylene (PP), while the second is polyethylene terephthalate (PET).

Polypropylene currently constitutes 21% of the global polymer production and 19% of production in Europe [31]. It is used to manufacture car parts, food packaging, and pipes. Many studies have shown the loss of storage stability of PP-modified bitumen [32]. On the other hand, the authors of paper [33] have obtained a different tendency. Some authors suggest adding a certain quantity of polyphosphoric acid to improve storage stability [27]. There are many promising studies suggesting that using PP can positively affect bitumen modification [14].

PET is a polymer used in most water bottles and food packaging. Its price limits its broader use. It has a high softening point, which substantially hinders its homogenisation when compared to other plastomers [14]. Nevertheless, many authors confirm its positive effect on bitumen properties [34]. Furthermore, there are studies confirming its positive effect on the permanent deformation resistance of mineral and bitumen mixes, especially in the dry mixing process [35]. It must be stated that the use of PET-type plastomer is a good choice for bitumen modification.

Based on the authors’ best knowledge, few comprehensive studies are published in relation to the use of plastomers, considering their modification, utilisation, and comparative data that will enable their use in road construction. Plastomer utilisation is one of the current global challenges related to environmental protection. Thus, it attracts many researchers to the topic of their implementation in road construction [36]. Due to the above, this paper contains information on bitumen and waste plastomer modification, with consideration given to a series of controlled variables, rheological effects, and the polymer-modified bitumen structure.

The available base of comprehensive research in the field of bitumen modification with plastomers is still limited, despite some implementations that take into account certain process factors during bitumen and plastomer modification. The conclusions contained in the papers cited earlier indicate that some plastomers can be used to modify bitumen for road and airport pavement construction [14], as they increase viscosity at operating temperatures [36]. In their extensive review of the state of the art, Polacco et al. [37] conclude that the use of plastomers requires more extensive studies incorporating multiple process factors. A similar position on this matter was adopted in [38], where the authors pointed out the need for continued research on the application of plastomers to bitumen as part of recycling. The literature cited above did not use experimental design assumptions to increase the representativeness of the results. The innovation introduced in the present article lies in the introduction of such a sampling scheme, which allows the identification of a regression model, free of systematic effects, that takes into account multiple process factors. This is a key element in the analysis of experimental results for obtaining optimal bitumen and plastomer compositions and evaluating the effects/phenomena that occur in the process of their modification.

## 2. Materials

### 2.1. Bitumen

The initial stage of testing featured steps aimed at a complex evaluation and selection of bitumen, the properties of which will be modified using selected polymers. Two different bitumen types were selected according to the adopted experiment design methodology. Their selection was dictated by the need to replicate different rheological conditions of the bitumen. Due to the above, the adopted bitumen types included “gel” with the penetration range of 20/30 and “sol-gel” with the penetration range of 70/100. Generally available commercial SBS-modified bitumen were also used in the analysis as reference. Bitumen PmB 45/80-55 was used as the reference bitumen. All bitumen were subjected to basic rheological testing. The test results with a 95% confidence interval are presented in Table 1.

Table 1 also contains the test result for bitumen PmB 45/80-55. The main objective was to refer the results obtained for plastomer-modified bitumen to the results obtained for this bitumen. The optimisation process was performed in a way that allowed the objective function to represent the behaviour of bitumen PmB 45/80-55 as much as possible.

### 2.2. Waste Plastomers

**Figure 1:**
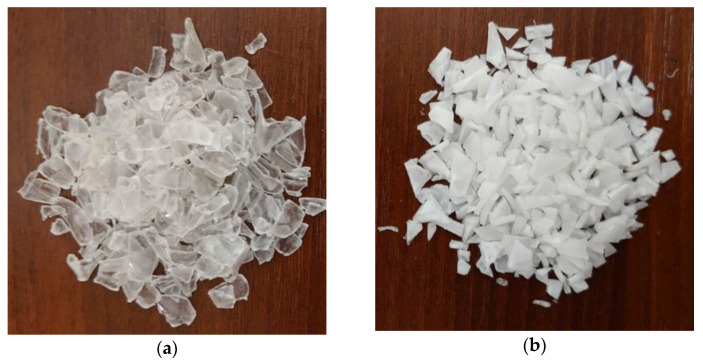
Samples of used plastomers: (**a**) PET (**b**) PP.

The modification of road bitumen properties was performed by the addition of two waste plastomers, i.e., PET (polyethylene terephthalate) and PP (polypropylene). The plastics used belong to the crystalline-structure thermoplastic materials group, whereas PET is characterised by a high softening point when compared to PP. An example of samples of plastomers (PP and PET) used in the tests, after being sieved through an 8 mm sieve, is shown in Figure 1.

Plastomers were provided by the manufacturer as a product of the shredding process. Following the suggestions proposed in paper [38], the supplied samples were sieved through an 8 mm sieve and then divided into two fractions separated by a 5.6 mm sieve to additionally evaluate the plastomer applicability without additional mechanical grinding. The waste polymer material used came from a single source. According to the plastomer supplier’s declarations, PP and PET were characterized by the features given in Table 2.

## 3. Methods

### 3.1. Microscope

The structural analysis of bitumen illuminated by the UV spectrum was conducted with the use of the AxioScope A.1 microscope. In the fluorescence microscope, light from the intense illuminator reaches the excitation filter through the heat filter (band-pass filter). Shortwave radiation reflects off a dichroic glass and is focused in the preparation by the lens. The preparation absorbs the shortwave radiation and emits longwave fluorescent radiation (Stoke’s law) that passes through a dichroic glass and is converted into an image by the lens. In the final stage, longwave radiation passes through a notch filter that only transmits longwave radiation emitted by the observed lens. The light emitted from the preparation is collected by the lens and, after passing through a filter set, is directed to an eyepiece. The wave transmission was within the range of 420 nm to 490 nm. The microscope used in the testing met the requirements of PN-EN 13632 [44].

### 3.2. Plackett–Burman Design

The Plackett–Burman design (P–B) is a special case of saturated orthogonal factorial designs. The P–B design is characterised by the fact that the number of experiments N is equal to the number of independent variables k increased by 1 [45]. This design is mainly used to select important independent variables from a set of input variables (tax extent reduction). The P–B design can be used to calculate the regression Equation (1):

(1) $$y=b_{0}+b_{1}\cdot x_{1}+\ldots +b_{n}\cdot x_{n}+\varepsilon$$

where, b_j_—regression coefficient (for j > 0), x_i_—independent variable (input factor), y—dependent variable, ε—estimation error.

It was demonstrated that the estimation of effects using the least squares method are independent from one another (orthogonal) and have the same and maximum accuracy in reference to number N. The experiment results variance y can be estimated based on additional designations. In this case, the entire experiment was replicated at least twice. The experiment’s replication was required, as random errors are unavoidable for such a high number of input variables. This design assumes the occurrence of omitted effects of interactions between the variables. If the effects occur, the model’s estimation error (1) will have high values, whereas the determination coefficient R^2^ will quickly be reduced.

The input variables assume values at two levels. These included min. and max. values for the quantitative variables, whereas the qualitative variables involved two states in at least the ordinal scale. The number of experiments in the P–B design for two levels is a product of 4, meaning that N is at least 8. Due to the above, the smallest plan that can be implemented must feature 7 independent variables. Such a plan was used in this test. The breakdown of variables and their respective quantities are presented in Table 3.

The preliminary determination of the ranges of values assigned to particular variables was based on literature analyses [47,48]. In the case of quantitative variables, the scope was determined to reflect high-speed and low-speed mixing. The plastomer content was limited to 5% to prevent the occurrence of excessive dynamic viscosity > 3 Pas at 135 °C [49]. Additionally, the impact of the plastomer’s fragmentation was considered in the experiment. Due to the above, the testing involved using material with granulation as supplied from the manufacturer (>5.6 mm) and following sifting of a certain fraction of waste polymer particles (<5.6 mm).

### 3.3. Blender Device Setup

**Figure 2:**
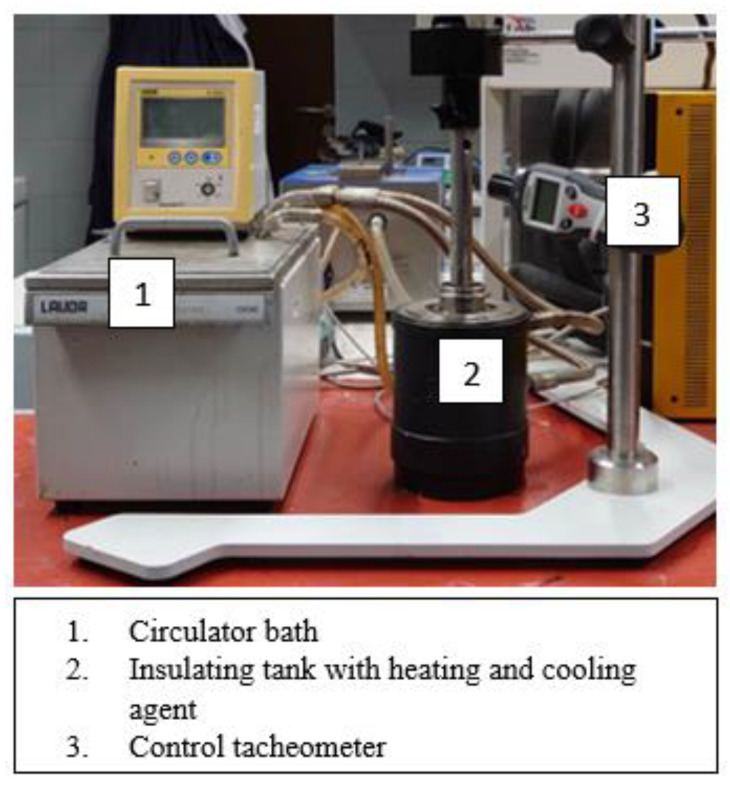
System for bitumen modification with plastomer.

The test setup for mixing bitumen and plastomer consisted of two components: a temperature control set and a blender. The temperature control system was based on the LAUDA E300 immersing circulator, which allowed the temperature to be maintained up to 200 °C. The blender had the ability to control the speed from 100 to 33,000 rpm (required in the experiment). As the controlling of the speed given by the blender relative to the actual value was a very important issue, an additional tachymeter was mounted to verify the blender actual speed before each test. An example of the equipment for bitumen modification with polymer is shown in Figure 2.

The system for mixing bitumen and plastomer allowed representing the case of low-speed and high-speed mixing of components with all the ranges of factors of the Plackett–Burman design given in Table 3.

### 3.4. MSCR Test Setup

**Figure 3:**
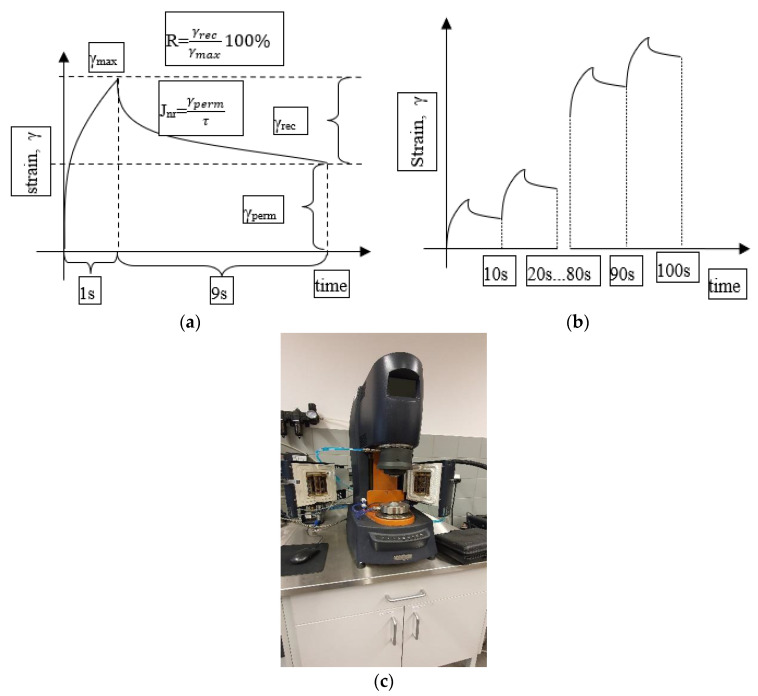
Execution principle of MSCR test; (**a**) one cycle scheme; (**b**) scheme of full test for 10 cycles; (**c**) Discovery Hybrid Rheometer HR-1 setup.

The viscoelastic polymer-modified bitumen was primarily analyzed on the basis of MSCR test. Multiple Stress Creep Recovery tests (MSCR) were performed at temperatures that cover potential maximum PG temperature. A total of 10 cycles of creep and recovery—1 s load and 9 s recovery—at 100 Pa was applied in the asphalt binder sample between the two parallel plates of the DSR (25.0 mm in diameter and 1.0 mm spacing between plates) followed by other 10 cycles at 3200 Pa [50]. The percentage of recovery (R) and nonrecoverable creep compliance (Jnr) were calculated for all cycles at 100 and at 3200 Pa, and the final results for both parameters correspond to the arithmetic average of the respective values’ difference between the non-recoverable creep compliances at two different stress levels (%). Asphalt binders were classified based on traffic volume, using the value of Jnr as a parameter. This parameter allows the evaluation of the resistance to rutting and it presents a good correlation with the mechanical tests on asphalt mixtures [51]. An exemplary course of the experiment is shown in Figure 3.

## 4. Results and Discussion

### 4.1. Basic Bitumen Properties

The testing of the impact of bitumen modification by selected plastomers started with basic tests. The tests were conducted on samples specified in the design presented in Table 3. The list of tests and the relevant standards are presented below:

- Softening point (T_R&B_) acc. to EN1427 [40],
- Penetration grade acc. to EN 1426 [39],
- Breaking point (acc. to T_Frass_) acc. to EN 12593 [41],
- Dynamic viscosity (η_135_) at 135 °C acc. to ASTM D4402 [43],
- Cohesion (including Elongation) acc. to EN 13703 [42],
- Penetration index (IP) acc. to EN 12591 [52].
- The results of the designations adopted in the experiment are presented in Table 4.

**Figure 4:**
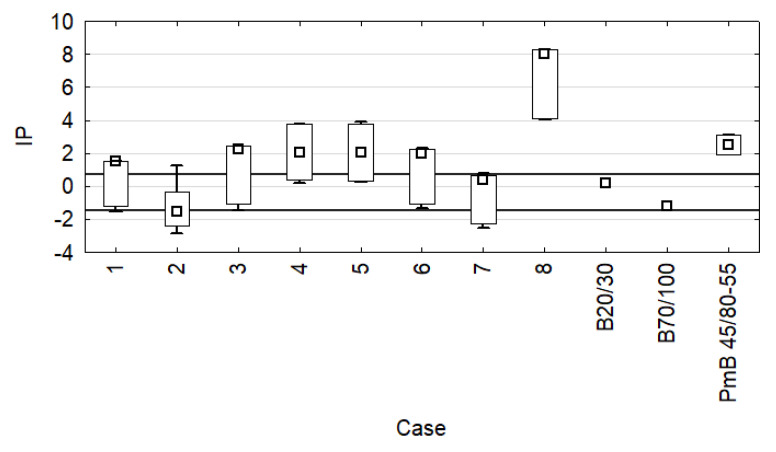
Designated penetration index values.

In Table 4, all results are presented along with their variability by specifying their confidence intervals of 95%. The IP value was calculated based on the penetration and softening point, to obtain information on the tested components’ thermal sensitivity. The IP results are presented in Figure 4.

The IP range for bitumen is usually from −3 for bitumen with high temperature sensitivity to approx. +7 for highly oxidised bitumen with low temperature sensitivity. It must be noted that variant 8 s (Figure 4) achieved IP = +6, which indicates that it has low temperature sensitivity. This also suggests the possibility of fatigue cracks in the mineral and bitumen mix made with this variant. The most temperature-sensitive bitumen was bitumen 70/100 and mixture 2 s containing PP. Furthermore, Figure 4 features the recommended IP range ∈ <+ 0.7; −1.5> for road bitumen in Poland. Two road bitumen (20/30 and 70/100) achieved values within the range. On the other hand, the vast majority of bitumen had values similar to the IP of bitumen PmB 45/80-55. It must be emphasised that this is only a measure of the bitumen’s thermal sensitivity, not a specification of its viscoelastic properties. The high IP of bitumen PmB 45/80-55 can be a result of the bitumen’s crosslinking caused by the elastomer, while, in the case of the plastomer-modified bitumen, the effect can be similar to the presence of a small filler in the bitumen.

### 4.2. Polymer Particle Distribution

The polymer particles’ quantitative analysis required digital image processing. For this purpose, the images collected during the observation of the polymer particles’ dispersion in the bitumen were subjected to digital processing with the use of a series of digital filters in the ImageJ program [53]. This allowed for the collection of a series of geometrical features that enabled an objective description of the polymer’s dispersion quality in the bitumen. The following three geometrical features were selected for the analysis based on the paper of Ralph et al. [54]:

- Surface area
- Roundness coefficient described by the following Equation (2)
  (2) $$roundness\;coefficient=4\cdot \pi \cdot \frac{Area}{Perimeter^{2}}$$
- largest particle size (major diagonal)

**Figure 5:**
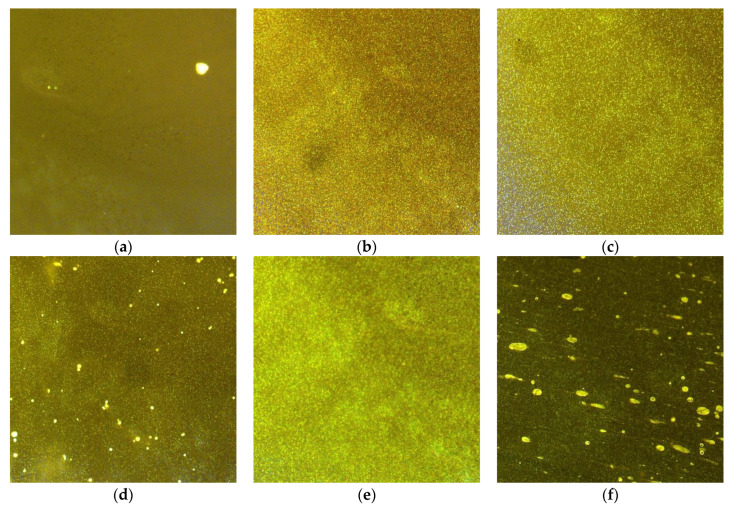
Microstructures of polymer-modified bitumen: (**a**) 2 s (**b**) 4 s (**c**) 6 s (**d**) 8 s (**e**) PMB 45/80-55 (**f**) PMB 45/80-65.

A selected result of sample observation (codes given in Table 4) in the epifluorescence microscope (high velocity variant) is presented in Figure 5.

**Figure 6:**
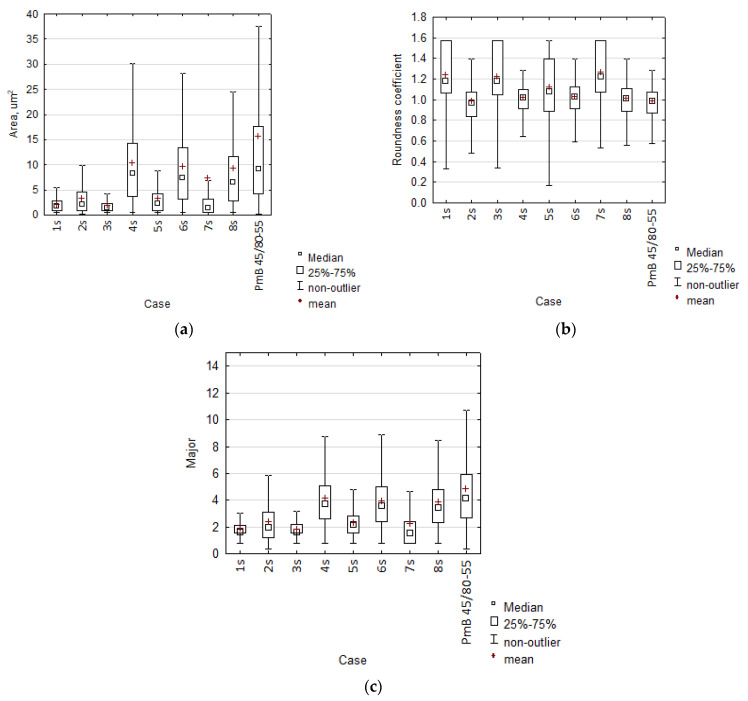
Quantitative analysis of the dispersion of plastomer particles in bitumen: (**a**) particle surface; (**b**) roundness coefficient; (**c**) largest particle size (Major diagonal).

It is necessary to note the differences in the dispersion structure and the homogenisation of bitumen modified with PP (Figure 5a,d) and PET (Figure 5b,c). The visual evaluation of the PP-modified bitumen looked more favourable than the PET-modified bitumen. The key issue was presumably the softening point of these plastomers. PET has a high softening point > 170 °C, which probably makes it difficult to obtain good bitumen-polymer homogenisation. A statistical analysis of the test results was conducted to objectively determine the polymer’s dispersion, considering the mixing conditions. A description of the statistical distribution of selected geometrical features is presented in Figure 6.

First, it is important to note that the surface area of the plastomer particles increases with high mixing speeds (Figure 6a). In addition, observations of the degree of particle roundness (Figure 6b) indicated that the polymer particle became rounder (>1.0) as the particle surface area decreased. The low value of the roundness coefficient observed in some cases was related to the fact that during high-speed mixing, some fine polymer particles aggregated strongly and eventually, the resulting particle acquired an ellipse-like shape with a very irregular lateral edge, suggesting its large specific surface area. Analysis of Equation (2) suggests that the value of the denominator increased rapidly in relation to the surface area, giving rise to the roundness coefficient value of <1.0. In the case of slow-speed mixing, usually less than 25% (first quartile) of the polymer particles had the roundness coefficient of <1.0.

When analysing the results presented on the plot in Figure 6c, it can be concluded that all plastomer-modified bitumen samples achieved an average particle size < 10 μm, which classifies them as having a fine-grained structure according to [44]. On the other hand, the particle dispersion structure in bitumen PmB 45/80-65 must be classified as medium-grained. The complex microstructure formation of plastomer-modified bitumen was subjected to a variance analysis. As a better illustration, the evaluation of the effects affecting the dispersion structure of polymer in bitumen was presented with the use of the Paretto plot for standardised values.

**Figure 7:**
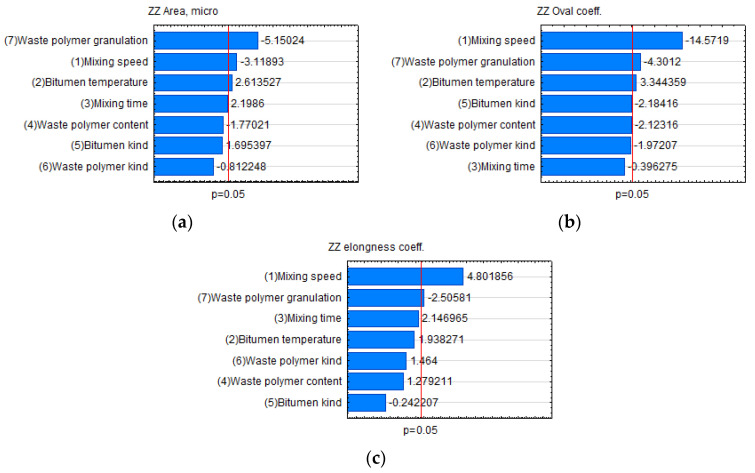
Quantitative analysis of the dispersion of plastomer particles in bitumen: (**a**) particle surface area; (**b**) roundness coefficient; (**c**) major diagonal.

The probability distribution of the dispersion of plastomer particles in bitumen did not deviate substantially from normal distribution. Due to the above, Figure 7 uses the linear variance analysis results and standardised effects evaluation (Paretto plots). This type of analysis enables a broader view of all factors that affect the modified bitumen’s structure. The mixing rate undeniably affected the microstructure. In the case of the roundness coefficient (Figure 7b), high mixing speed prevented polymer particles from becoming round. The polymer’s granulation also played an important role. It turned out that adding polymer with granulation > 5.6 mm resulted in particle surface area reduction (negative growth) and that the particles did not become elongated, as in the case of bitumen PMB 45/80-55. Therefore, it can be presumed that the large particle size hindered aggregation and possible crosslinking of the bitumen structure caused by the plastomer. Another important factor was the mixing temperature. A change in mixing temperature was statistically significant for the Area and Oval coefficient variables. The increasing specific surface area can be attributed to further plastomer polymerisation, which was correlated with the temperature. It is interesting that the polymer type and quantity did not substantially affect the particle dispersion in the bitumen, nor did the bitumen type with a specific penetration range. Therefore, to achieve the desired microstructure, it is necessary to pay attention to the components’ mixing rate, the plastomer granulation, and mixing temperature when planning the process.

### 4.3. MSCR Measurement

**Figure 8:**
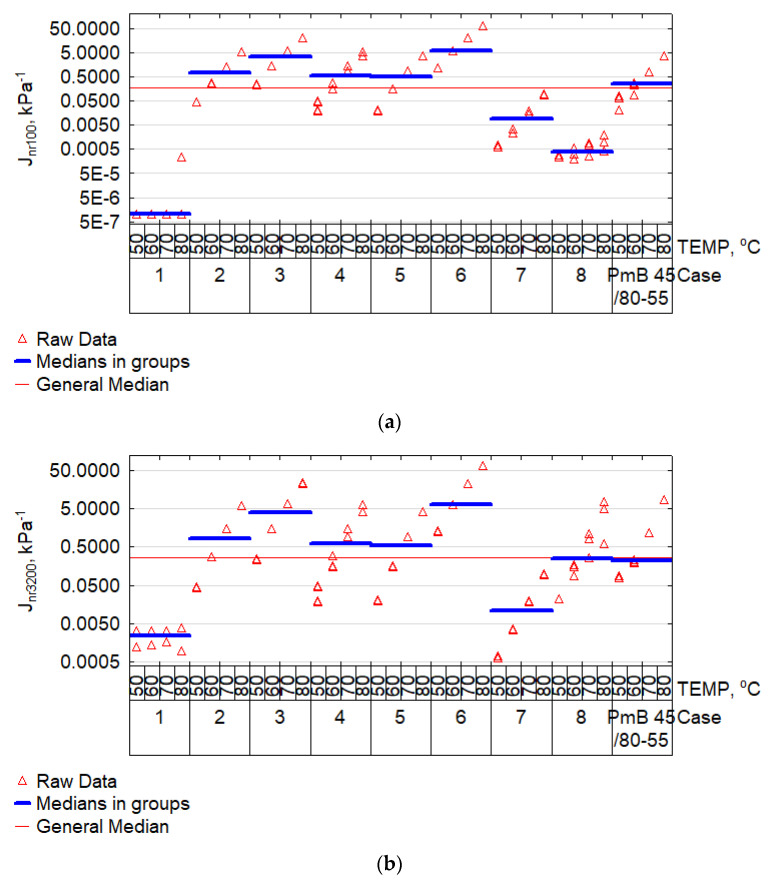
Plot of creep compliance Jnr for shear stress: (**a**) 100 Pa (Jnr_100_); (**b**) 3200 Pa (Jnr_3200_).

**Figure 9:**
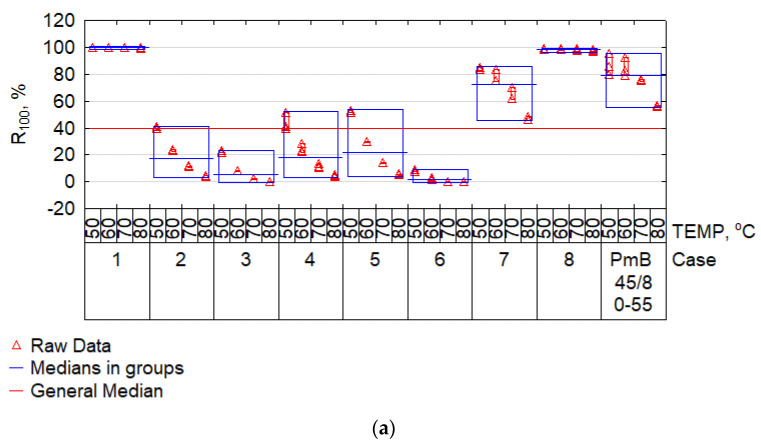

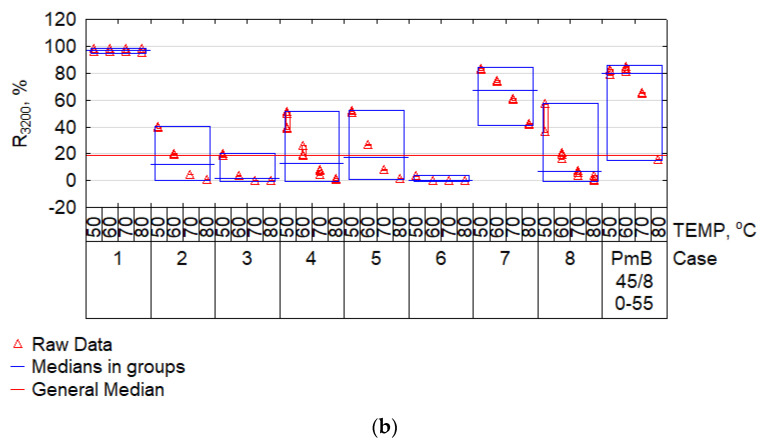
Plot of elastic recovery R for shear stress: (**a**) 100 Pa (R_100_); (**b**) 3200 Pa (R_3200_).

The MSCR test was especially important, as it enabled the determination of the impact of the given bitumen on the predicted rutting resistance of mineral and bitumen mixes. It is especially important for the impact of high temperatures on mma. The bitumen’s creep testing was subject to the requirements of EN 16659 [55] with the use of the dynamic shear rheometer. The testing covered all modified bitumen samples with composition imposed by the Plackett–Burman design (Table 3). The results of designating creep compliance (Jnr) and elastic recovery (R) at stress 100 Pa and 3200 Pa and three temperatures: 50 °C, 60 °C, 70 °C are presented in the plots of Figure 8 and Figure 9.

**Figure 10:**
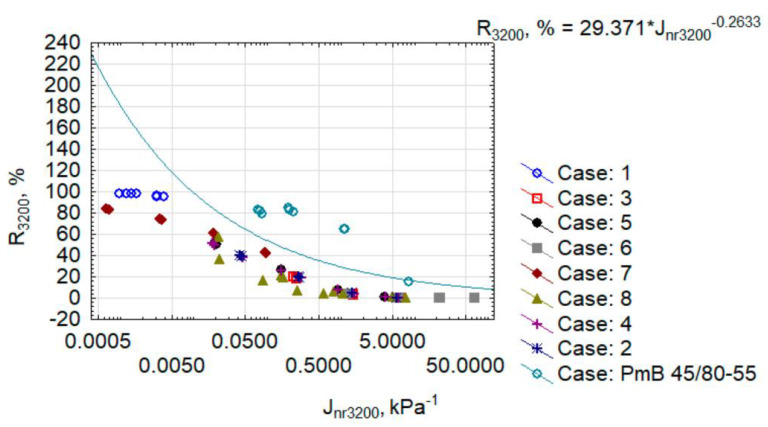
Creep compliance vs recovery.

Modified bitumen PmB 45/80-55 was added as a reference material to the results presented in Figure 8 and Figure 9. The median was used as the central value for comparisons. It must be noted that a comparable results proportion distribution was maintained when analysing the results in Figure 8a,b. A sudden increase of compliance Jnr and a reduction of R_3200_ was only observed in case no. 8 when comparing the Jnr and R results at shear stress of 100 Pa and 3200 Pa. By far the lowest compliance was achieved by cases no. 1, 7, and 8, i.e., those containing PP. The result for case no. 8 correlated with an extremely high IP value (Figure 4). For these cases, the compliance J_nr3200_ was below the median for the full set and was similar to the median of the results for modified bitumen PmB 45/80-55 (reference material). The highest average compliance was achieved by cases no. 3 and 6, i.e., those containing PET mixed with bitumen 70/100. The high fine-grained PP content, represented by case no. 1, resulted in a very low J_nr3200_ (Figure 8b) and very high R_3,200_ (Figure 9b). Such a combination of parameters can suggest an elastic and brittle state, which is unfavourable at low temperatures. Most cases oscillated around the median of J_nr3200_ = 0.5 kPa^−1^. According to AASHTO M 332 and AASHTO T 350, such bitumen can be classified for use in extremely heavy traffic (>30 million axles (ESAL) and vehicle parking (<20 km/h)), i.e., as in the case of SBS-modified bitumen. Nevertheless, in terms of R_3200_, plastomer-modified bitumen were substantially less effective than bitumen PMB 45/80-55. As an exception, case no. 7, containing fine-grained PP modified with bitumen 20/30 at high temperatures, allowed us to obtain an R_3200_ comparable with the reference bitumen at a substantially lower compliance J_nr3200_. To take a more complex look at the obtained results, in terms of the effectiveness of plastomer modification, the results were projected as J_nr3200_-R_3200_ and presented in Figure 10.

**Figure 11:**
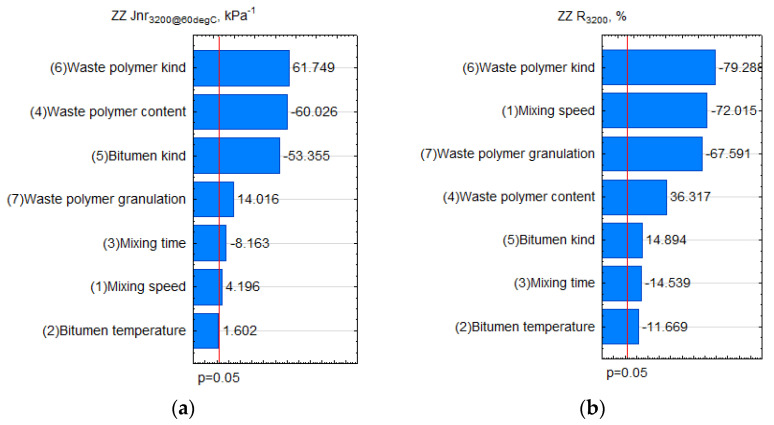
Pareto plot of standardised effects: (**a**) J_nr3200_, (**b**) R_3200_.

The presented cases derive from the MSCR test conducted at temperatures: 50 °C, 60 °C, and 70 °C. It must be noted that all results are below the conventional line separating modified bitumen with an acceptable degree of elastomer modification (acceptable elastomeric polymer). Among those, only bitumen PMB 45/80-55, modified with the SBS elastomer, was within the acceptable zone (area above the conventional line). In the collection of plastomer-modified bitumen, the most promising was the composite of cases no. 1 and 7. Unfortunately, case no. 1 was too rigid, as specified above. However, when analysing the entries of AASHTO T350 [50], R above the conventional line suggests the presence of elastomer-modified bitumen. Nevertheless, Morales et al. [56] point to a certain imperfection of the R parameter’s interpretation, which indirectly correlates with the rutting resistance of mineral and bitumen mixes. Due to the above, the fact that the results of case no. 7 are slightly below the line designated by the threshold function does not mean that the bitumen must be disqualified as modified bitumen. It only means that the answer during the MSCR test can be slightly different from other elastomer-modified bitumen. When observing the nature of the impact of the variables on Jnr and R, no direct correlation between them was recorded. This raises the question of which variables affect the results and how they affect the given feature. The variance analysis was used for this purpose. The variance analysis was conducted at the significance of 5%. Its results are best represented by the Pareto plot of standardised effects, presented in Figure 11.

When analysing Figure 11a, it turned out that temperature did not substantially affect the feature J_nr3200_. On the other hand, this feature was affected significantly by the polymer content, type, and basic bitumen type. It is necessary to consider the plastomer’s pour point and glass transition point. It was observed that the higher the polymer content and the harder the bitumen, the lower the J_nr3200_ value became. On the other hand, the use of a plastomer with a high softening point (PET) substantially increased the compliance, which could be a symptom of low homogenisation between the bitumen and PET, thereby, a symptom of possibly low composite storage stability. In reference to J_nr3200_, it was favourable to increase the mixing rate for a mixing time shorter than 180 min. Based on our own observations, the extended high-speed mixing time caused plastomer particle coagulation and segregation of components.

The impact of the process variables was slightly different for R_3200_. The waste polymer’s type was again the most important factor. As in the case of feature J_nr3200_, PP’s lower pour point allowed for obtaining increased elastic recovery R_3200_. The low mixing rate was also favourable for the feature’s elastic recovery. The initial PP fragmentation degree was probably responsible for this observation. The fragmentation degree also turned out to be an important variable. According to the plot (Figure 11), the smaller the plastomer addition with a mixing time < 180 min, the higher (more favourable) the R_3,200_ obtained.

In summary, to obtain plastomer-modified bitumen with the desired properties Jnr and R, it is necessary to perform optimisation, as the mixing process variables have varied impact and effectiveness. Furthermore, it is also necessary to determine the degree to which the Boltzman superposition principle applies to the plastomer-modified bitumen behaviour description within the linear viscoelasticity (LVE) limits. The bitumen testing temperatures: 50 °C, 60 °C, and 70 °C are probable in the road surface. The behaviour of plastomer-modified bitumen, in accordance with the LVE model, will ensure energy dissipation minimisation and, more importantly, will minimise the rate of permanent deformation accumulation on the road surface.

### 4.4. Viscoelastic Properties of Selected Polymer-Modified Bitumen

The tested waste polymer-modified and SBS-modified bitumen underwent a detailed rheological analysis. Its purpose was to describe the strain during creep, using the MSCR test and utilising the generalised Kelvin–Voigt model (Prony series). The good match of experimental data with this model suggests that the bitumen are within the linear viscoelastic (LVE) limits. The discrepancy in the model’s results when compared to the experiment can point to non-linear behaviour τ-γ and provide information about the occurrence of substantial plastic strains. The (shear) relaxation function G is described with the following equation [57,58] (3):

(3) $$G\left(\psi^{t}\right)=G_{0}\left(1+\overset{n}{\underset{i=1}{\sum }}g_{i}\left[1-e^{\left(-\lambda_{i}\psi^{t}\right)}\right]\right)$$

where, $G_{0}$—instantaneous compliance, $g_{i}$—i-th compliance corresponding to the next Kelvin–Voigt element, $\lambda_{i}$—i-th retardation time, $\psi^{t}$—reduced time $\psi^{t}=\frac{t}{a_{\sigma }}$

In the paper, the mastic samples were subjected to pure shearing as part of controlled shear stress. In the analysis, it was assumed that the creep function within the LVE limits will be best represented by a generalised model, consisting of series of parallel Kelvin elements and a single Hooke element. Based on earlier analyses, it was decided that a setup of five Kelvin elements (*n* = 5) and a single Hooke element is sufficient to correctly describe the bitumen’s strain changes.

The estimation of the model’s parameters required the use of the non-linear least squares method to minimise the objective function at the pre-determined initial values. For this purpose, a complex block script, aimed to seek the minimum objective function, was developed in the MathCad program, using the Quasi-Newton method. In order to avoid issues related to the correct determination of the initial values, the MCalibration^®^ program, utilising an implemented set of solvers, was used for verification purposes [59].

The tests were conducted for several temperature levels (50 °C, 60 °C, 70 °C), with the aim of reproducing the behaviour of waste mixtures to the highest degree possible. Therefore, in order to take into account the temperature effect to build the dynamic module’s master curve, it was necessary to use the time–temperature superposition principle (TTSP), which combines the load frequency and temperature in the form of the horizontal temperature shift coefficient $\alpha_{T}$. The following form of the WLF equation was used for this purpose (4) [60].

(4) $$log\alpha_{T}=\frac{C_{1}\cdot \left(T-T_{0}\right)}{C_{2}+\left(T-T_{0}\right)}$$

where, C_1_, C_2_—experimental coefficients, T_0_—reference temperature, T—test temperature.

**Figure 12:**
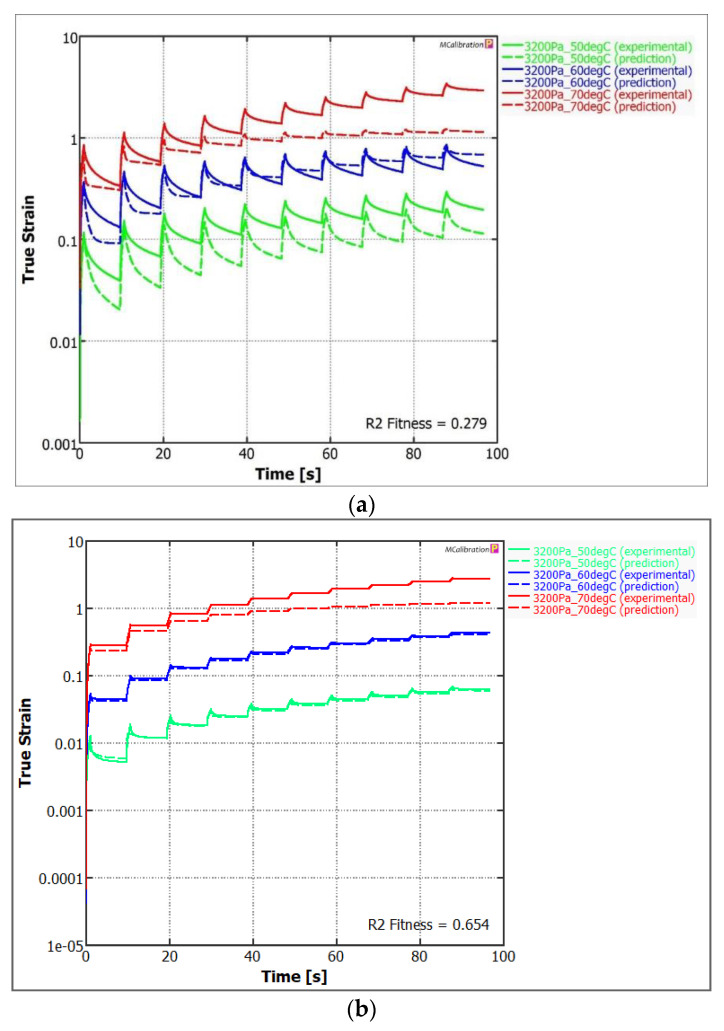
Comparison of the match between the generalised Maxwell model and the experimental results of cyclic strain at 100 Pa and 3200 Pa acc. to the MSCR [55]: (**a**) PmB 45/80-55; (**b**) case 8 s.

The model’s quality of matching with the experimental data was determined using two qualitative measures, i.e., the modified determination coefficient R^2^ and standardised root mean square error RMSE [61]. The graphic interpretation of matching the generalised Maxwell model to selected modification cases is presented in Figure 12.

The results of identification of the LVE model’s (generalised Maxwell model) parameters are presented in Table 5.

It must be noted that the plastomer-modified bitumen’s relaxation capacity differed depending on the case. When observing examples of representation of results in the MSCR test in Figure 12a,b, the model’s matching for case no. 8, based on the determination coefficient R^2^, was <0.7. This is a satisfactory value, which indicates that the bitumen’s creep is in accordance with the LVE model. The share of the viscous part in the modified bitumen’s strain was, therefore, small in each load cycle [62]. Furthermore, the deviation from the LVE model suggests that the stress level can contribute to the modified bitumen’s strain. In the case of bitumen PmB 45/80-55, the matching quality was low < 0.3. Nevertheless, it was possible to notice a high strain under load and during the bitumen sample’s relief. Taking into account the SBS’s rheological characteristics, it must be assumed that the hyperelastic model [63,64] would be more adequate in this case than the proposed generalised Maxwell model.

The breakdown of the parameters’ identification in the generalised Maxwell model is presented in Table 5. For cases no. 3 and 6, the relaxation function matching was not satisfactory. A high strain was observed at >60 °C. This was probably a result of disturbances in the bitumen’s structure caused by the stress that turned out to be critical in these cases. They included modifications of soft road bitumen 70/100, using PET-type plastomer. The best results were achieved by bitumen modified with fine-grained PP (cases no. 1 and 7), but also by PET-modified bitumen at higher temperature (case no. 5). Among the analysed cases, it turned out that the modification of soft bitumen 70/100 with greater polymer quantity of 5% allowed us to obtain satisfactory results, as in the modification of bitumen 20/30 with smaller plastomer quantity of 3%. Therefore, the bitumen composition and the aromatic fractions quantity are as important as in the bitumen modification by plastomer [16].

Further important information that is derived from the generalised Maxwell model is the ability to compare the intact (instantaneous) structure’s rigidity. The highest values were achieved by bitumen no. 1 and 7, i.e., those containing fine-grained PP < 5.6 mm and those modified at the mixer’s low mixing rate. These also included bitumen with J_nr3200_ below the median and with the highest elastic recovery R_3200_ among other plastomer-modified bitumen. The Go value (instantaneous compliance in formula (3)) for this bitumen was closest to the value achieved by the elastomer-modified bitumen PmB45/80-55. The obtained MSCR results are in line with the conclusions drawn following the generalised Maxwell model’s matching process. Due to the above, there is a certain solution, in the form of selecting specific process factors, which allows us to obtain plastomer-modified bitumen that will have properties similar to the elastomer-modified bitumen in terms of the rheological characteristics.

### 4.5. Optimisation Process

The optimisation process involved the determination of the regression models (1) based on the Plackett–Burman elimination design. The optimisation is approximate due to the inability of implementing the non-linear model in the P–B elimination design. Nevertheless, it provides certain information on the best configuration of bitumen and plastomer mixing parameters, because it considers a large number of variables that control bitumen modification using plastomer. The quantitative evaluation of the main effects using the ANOVA linear model was aimed at determining whether it is reasonable to search for the essential test object function that described all dependent variables (measured variables). In effect, it was concluded that at least one of the input variables had a substantial impact on the input variables’ variability. Due to the above, it was decided to identify the test object function parameters. Table 6 below presents the results of matching the linear model with formula (1). Building a regression model that would take into account qualitative and quantitative features required using the Generalised Linear Model algorithms [65]. The results of identification of the model’s parameters based on the experimental data are presented in Table 6.

It is necessary to point out the high significant coefficient R^2^, at an acceptable RMSEE, was in the range from 0.66 to 0.99. The last stage of analysis, i.e., the modification process optimisation, has commenced based on the identified significant mathematical regression models. The purpose of the optimisation, taking into account the pre-determined set of criteria, was to determine the most suitable configuration of the input variables to obtain a satisfactory output in the form of plastomer-modified bitumen [67,68]. Criteria were defined for this purpose and their results are presented in Table 7.

**Figure 13:**
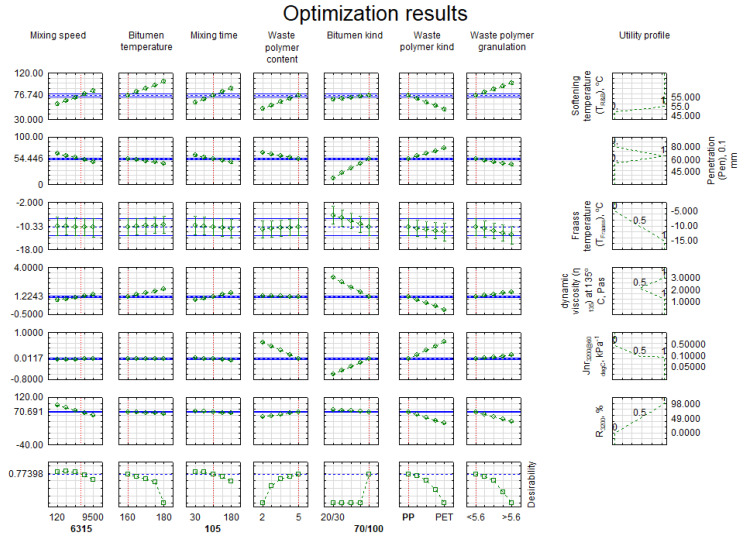
Results of the search for the optimal bitumen and plastomer mixing solution.

The main objective was to obtain a bitumen with high T_R&B_ similar to that of bitumen PmB 45/80-65, and free of aggregate coating issues at high temperatures (η_135_). Furthermore, the plastomer-modified bitumen should demonstrate low penetration and low breaking point (>−5 °C). In the case of penetration, the recommended range is from 45 × 0.1 mm to 80 × 0.1 mm. In effect, the optimisation resulted in the utility function (objective function), which, in essence, was a geometric mean [69]. The optimisation result is presented in Figure 13.

It must be noted that the utility function amounted to 0.78. This meant that above-average material properties were achieved relative to the criteria presented in Table 7. It turned out that obtaining material with these properties requires using a mixing rate of less than 9500 rpm, i.e., approx. 6000 rpm, and the mixing time should be approx. 105 min. According to previous observations, it is necessary to use 5% of PP-type plastomer with granulation <5.6 mm and use it to modify bitumen 70/100. The breakdown of results for all features is presented in Table 8.

In addition, a single additional composition was prepared, following the entire experiment for the optimal configuration derived from the P–B model, aimed at verifying the results obtained in the search for the optimal solution. The validation results are designated as “Experiment” in Table 8. It must be noted that the obtained validation results are very similar to the results obtained during optimisation, based on mathematical models (Figure 13). Unfortunately, the optimal solution is very close to the conventional limit designated in the aforementioned AAHTO T350 standard on the quality of modification of elastomer-modified bitumen. Nevertheless, the result is below the conventional limit line, as in Figure 10. The obtained optimal solution provides a modified bitumen that was substantially less creep-compliant than bitumen 

PmB45/80-55 and had favourable viscosity at 135 °C. On the other hand, it had a slightly higher breaking point and cohesion energy of 0.82 J/cm, which was lower than the threshold value of 2 J/cm, according to the Polish national requirements. It must be recalled that, according to the results in Table 1, road bitumen had negligible cohesion energy. Therefore, the high cohesion result for the plastomer-modified bitumen allows us to conclude that a properly arranged modification process can allow us to obtain bitumen with at least minimum properties as in PmB45/80-55. Due to the above, the long-term perspective assumes the continuation of tests on the improvement of cohesion at the waste polymer-bitumen contact limit by using, e.g., polyphosphoric acid [70]. In summary, due to its adequate viscosity at high temperature, the produced plastomer-modified bitumen will contribute to the good aggregate coating, while its low susceptibility in high temperatures will limit the strains in the bitumen surface at a satisfactory Frass temperature result.

## 5. Conclusions

The following conclusions were formulated based on the conducted tests and the analyses related to the modification of bitumen using waste plastomer:

1. The dispersion structure of waste plastomer particles in the bitumen matrix relies primarily on the mixing speed, plastomer particle size, and mixing temperature. Slow mixing resulted in the formation of more particles with a rounded shape than in the case of high-speed mixing. Almost all bitumen and plastomer mixtures obtained a fine-grained structure with a particle size < 10 μm. Therefore, it is expected that the required stability of bitumen storage will be maintained.
2. Soft bitumen (70/100) can be effectively modified with smaller plastomer quantities than hard bitumen (50/70). The same rule applies as in bitumen modification using elastomer.
3. Bitumen modified with fine-grained PP at a low mixing rate allows us to obtain low creep compliance (J_nr3200_) and high elastic recovery (R_3200_), similar to the elastomer-modified bitumen.
4. The use of bitumen modified with fine-grained PP allows us to obtain a similar instantaneous compliance module (Go) to the reference elastomer-modified bitumen.
5. The use of PP enabled achieving a satisfactory result of bitumen modification compared to the application of PET over the given experimental range. As a result, the optimization of the bitumen 70/100 modification with PP made it possible to obtain a mixture with rheological properties very similar to those of bitumen PmB 45/80-55. The validation test confirmed this result.
6. The best result was obtained by applying 5% fine-grained PP plastomer to bitumen 70/100 at 160 °C at a mixing speed of about 6300 rpm for 105 min. The obtained characteristics indicate an increase of cohesion relative to bitumen 70/100 while maintaining the properties at low (Fraass breaking point) and high service temperatures (T_R&B_) compared to elastomer-modified bitumen. In addition, this set of mixing process parameters provides the optimal viscosity required for proper aggregate coating (η < 2 Pas).

In the near future, the authors are planning to continue the tests on modifying bitumen with plastomers using modifiers that improve the storage stability. Further research is also envisaged with the focus on a promising PET plastomer that requires broadening the experiment’s design.

## Figures and Tables

**Table 1:** Results of road paving bitumen testing.

Feature	Neat Bitumen		Referenced Bitumen	Standard
	20/30	70/100	PmB 45/80-55	
Penetration at 25 °C, 0.1 mm	27.4 ± 2.1	91.5 ± 3.1	66.6 ± 2.8	PN-EN 1426 [39]
Softening point T_R&B_, °C	61.3 ± 1.2	44.7 ± 0.7	63.7 ± 2.5	PN-EN 1427 [40]
Fraass breaking point, °C	−10.4 ± 2.2	−15.4 ± 2.0	−17.7 ± 1.8	PN-EN 12593 [41]
Elongation at 5 °C, cm (declared)	143	316	>400	PN EN 14023 [42]
Cohesive energy, J/cm^2^	-	0.066 *	7.4	PN EN 14023
Viscosity at 60 °C, Pas	3624	112		ASTM D 4402 [43]
Viscosity at 90 °C, Pas	96.4	7.6		
Viscosity at 135 °C, Pas	1.43 ± 0.01	0.32 ± 0.01	3.32 ± 0.01	

*—result within the ductility meter’s detection limit.

**Table 2:** Plastomers basic properties.

Feature	PP	PET
Melting point, °C	165	256
Glass transition point, °C	−10	75
Density Mg/m^3^	0.91	1.32
Melt flow index (g/10 min)	0.22	35.1

**Table 3:** Variables in the Plackett–Burman experimental design [46].

No.	Variables	Type	Unit	Code	Low Level/ Level One	High Level/ Level Two
1.	Mixing speed	quantity	rpm/min^−1^	A	120	9500
2.	Mixing temperature	quantity	°C	B	160	180
3.	Mixing time	quantity	min.	C	30	180
4.	Waste polymer content	quantity	%	D	2	5
5.	Bitumen type	quality		E	20/30	70/100
6.	Waste polymer type	quality	-	F	PP	PET
7.	Waste polymer granulation	quality	-	G	<5.6 mm	>5.6 mm

**Table 4:** Results of modified bitumen properties designation based on the Plackett–Burman design.

Case	Independent Variable							Dependent Variable					
	A	B	C	D	E	F	G	T_R&B_	Penetration	Breaking Point	Cohesion	Elongation	Dynamic Viscosity
	rpm/min^−1^	°C	min	%	-	-	-	°C	0.1 mm	°C	J/cm^2^	mm	Pas
1 s	120	160	30	5	70/100	PP	<5.6	53.7 ± 3.7	72.6 ± 0.8	−9.9 ± 2.2	0.134	283 ± 161	0.53 ± 0.01
2 s	9500	160	30	2	20/30	PP	>5.6	78.8 ± 7.5	28.2 ± 0.8	−10.7 ± 0.4	0.000	84 ± 14	2.2 ± 0.02
3 s	120	180	30	2	70/100	PET	>5.6	54.4 ± 4.5	86.7 ± 1.8	−12.7 ± 2.3	0.098	290 ± 146	0.45 ± 0.01
4 s	9500	180	30	5	20/30	PET	<5.6	59.1 ± 5	21.3 ± 1.7	−9.7 ± 1.3	0.000	97 ± 16	3.73 ± 0.1
5 s	120	160	180	5	20/30	PET	>5.6	56 ± 4.5	19 ± 3.5	−10.3 ± 1	0.000	67 ± 16	3.32 ± 0.01
6 s	9500	160	180	2	70/100	PET	<5.6	55.2 ± 4.3	76.6 ± 2.5	−11.9 ± 1.7	0.117	481 ± 270	0.53 ± 0.01
7 s	120	180	180	2	20/30	PP	<5.6	78.6 ± 7.6	28 ± 1	−8.4 ± 1	0.000	146 ± 52	2.3 ± 0.16
8 s	9500	180	180	5	70/100	PP	>5.6	117.8 ± 20	21.4 ± 2.5	−10.7 ± 0.9	0.000	70 ± 2	2.89 ± 0.07

**Table 5:** Generalised Maxwell Model parameters.

Parameters	1	2	3 *	4	5	6 *	7	8	PmB 45/80-55
G_o_, kPa	585.6	446.8	255.6	300.3	264	1.4	665.3	331.7	811.1
g_1_	0.61	0.32	0.7	0.45	0.68	0.43	0.79	0.2	0.59
t_1_, s	0.003	0.03	0.008	0.26	0.04	0.61	0.31	0.07	0.002
g_2_	0.39	0.21	0.39	0.49	0.4	0.11	0.39	0.19	0.38
t_2_, s	7 × 10^−3^	0.003	0.007	0.075	0.008	1.04	0.07	0.07	1 × 10^−4^
g_3_	0.01	0.188	0.005	0.003	0.01	0.06	0.04	0.09	0.007
t_3_, s	0.21	0.07	0.72	5.93	0.88	0.32	0.19	0.08	0.27
g_4_	0.08	0.17	0.014	0.12	0.013	0.09	0.017	0.01	0.09
t_4_, s	0.001	0.035	0.0008	0.02	0.03	0.78	0.004	0.06	0.003
g_5_	0.04	0.001	1 × 10^−6^	0.001	0.003	9 × 10^−4^	0.06	0.07	0.004
t_5_, s	28.8	8.54	6.4	2.28	8.33	1.43	4.56	0.38	4.34
R^2^ RMSEE,%	0.83 28.9	0.66 20.3	0.44 32.6	0.64 23.6	0.84 12.4	- 74.8	0.68 14.7	0.65 17.9	0.28 31.3
WLF									
C_1_	1.14	3.7	1.94	3.7	2.71	1.96	4.56	13.4	2.8
C_2_	29.5	50.3	49.3	52.2	38.1	37.5	57.7	166.7	34.4

*—extremely high compliance for 3200 Pa at 70 °C.

**Table 6:** Linear regression model parameters.

Variable	Penetration 0.1 mm	T_R&B_^a^ °C	Fraass °C	J_nr3200_ kPa^−1^	R_3200_ %	η_135_ Pas
Intercept	44.3042	67.8234	−10.7833	−80.4939	7225.412	1.9926
(1) mixing speed	−8.3708	13.1487	−0.0083	0.0000	−0.004	0.2657
(2) mixing temperature	−5.1292	12.7188	0.1333	0.0009	−0.280	0.3476
(3) mixing time	−7.8792	13.1694	−0.7417	−0.0006	−0.047	0.3430
(4) waste polymer content	−7.0542	12.8279	0.6583	−0.2145	5809	−0.0140
(5) bitumen type	−19.6958	−4.1272	1.7167	−0.5719	7.146	0.8959
(6) waste polymer type	10.6958	−13.4365	−0.5917	0.6618	−38.044	−0.6251
(7) waste polymer granulation	−5.8708	12.7481	−1.1333	0.1502	−32.431	0.2211
R^2^ [66]	0.98	0.98	0.66	0.99	0.98	0.99
RMSEE [61]	2.9	4.4	2.1	0.02	1.0	0.053

^a^—values marked in red were determined as significant.

**Table 7:** Utility profile ranges adopted for optimisation (criteria).

Level	Penetration 0.1 mm	T_R&B_ °C	T_Fraass_ °C	J_nr3200_ kPa^−1^	R_3200_ %	η_135_ Pas
Low (value 0)	45 ÷ 80	<55	>−5	0.5	<30	>3
High (value 1)		>80	<−15	0.05	>80	<1

**Table 8:** Results of the optimal solution validation.

Feature	Optimisation Results: Mixing Speed = 6315 rpm Mixing Temperature = 160 °C Mixing Time = 105 min. Waste Polymer Content = 5% Bitumen Type = 70/100 Waste Polymer Type = PP Waste Polymer Granulation ≤ 5.6 mm		PMB45/80-55
	Optimal Results from Model (Theoretical)	Experimental Results from Optimal Setting (Validation)	Experiment (PMB45/80-55 Referenced)
Penetration, 0.1 mm	77	79	67
T_R&B_, °C	55	56	64
T_Fraass_, °C	−10.3	−14.1	−17.6
J_nr3200_, kPa^−1^	0.02	0.06	0.21
R_3200_, %	70.7	56	83
η_135_, Pas	1.2	1.0	2.4
AASHTO T350	not pass	not pass	pass
Energy, J/cm	-	0.82	7.4

## Data Availability

Data available on request from the corresponding author.
